# Anti-Biofilm Activity of Carnosic Acid from *Salvia rosmarinus* against Methicillin-Resistant *Staphylococcus aureus*

**DOI:** 10.3390/plants12213679

**Published:** 2023-10-25

**Authors:** Valeria Iobbi, Valentina Parisi, Giulia Bernabè, Nunziatina De Tommasi, Angela Bisio, Paola Brun

**Affiliations:** 1 Department of Pharmacy, University of Genova, Viale Cembrano 4, 16148 Genova, Italy; valeria.iobbi@edu.unige.it; 2 Department of Pharmacy, University of Salerno, Via Giovanni Paolo II 132, 84084 Salerno, Italy; vparisi@unisa.it (V.P.); detommasi@unisa.it (N.D.T.); 3 Department of Molecular Medicine, University of Padova, Via Gabelli 63, 35121 Padova, Italy; giulia.bernabe@unipd.it (G.B.); paola.brun.1@unipd.it (P.B.)

**Keywords:** *Salvia rosmarinus*, carnosic acid, quorum sensing, MRSA, anti-virulence, biofilm

## Abstract

The *Salvia rosmarinus* “Eretto Liguria” ecotype was studied as a source of valuable bioactive compounds. LC-MS analysis of the methanolic extract underlined the presence of diterpenoids, triterpenoids, polyphenolic acids, and flavonoids. The anti-virulence activity of carnosic acid along with the other most abundant compounds against methicillin-resistant *Staphylococcus aureus* (MRSA) was evaluated. Only carnosic acid induced a significant reduction in the expression of *agrA* and *rnaIII* genes, which encode the key components of quorum sensing (QS), an intracellular signaling mechanism controlling the virulence of MRSA. At a concentration of 0.05 mg/mL, carnosic acid inhibited biofilm formation by MRSA and the expression of genes involved in toxin production and made MRSA more susceptible to intracellular killing, with no toxic effects on eukaryotic cells. Carnosic acid did not affect biofilm formation by *Pseudomonas aeruginosa*, a human pathogen that often coexists with MRSA in complex infections. The selected ecotype showed a carnosic acid content of 94.3 ± 4.3 mg/g. In silico analysis highlighted that carnosic acid potentially interacts with the *S. aureus* AgrA response regulator. Our findings suggest that carnosic acid could be an anti-virulence agent against MRSA infections endowed with a species-specific activity useful in multi-microbial infections.

## 1. Introduction

*Salvia rosmarinus* Spenn. (Lamiaceae) [1,2,3] (“rosemary”) is an aromatic evergreen shrub native to the Mediterranean region, and it has been used for centuries for its culinary, medicinal, and aromatic properties [4]. Several Italian ecotypes have been studied for their possible exploitation [5,6,7]. “Eretto Liguria” is a rosemary ecotype widely used in northwestern Italy by growers for cuttings and marketing. The identification of possible uses of waste biomass from agricultural cultivation and harvesting practices is crucial to developing the local economy more effectively [8,9]. Indeed, various bioactive compounds of *S. rosmarinus* extracts [10,11,12,13], including essential oils, phenolic compounds, flavonoids, and other secondary metabolites, are endowed with antibacterial properties [14,15,16,17,18,19,20,21], and, due to the high chemodiversity of the species [7,22,23,24,25], local rosemary ecotypes have been considered as sources of bioactive extracts [26,27,28]. Several studies reported that the antibacterial activity is mainly related to their content of phenolic abietane diterpenes, such as carnosic acid (CA) and its derivatives, and phenolic acids, such as rosmarinic acid [29,30]. CA and carnosol are the major antioxidants and antibacterial compounds in rosemary extracts [31,32,33]. They are used as natural ingredients in various products, including dietary supplements, cosmetics, and food products. Moreover, they are considered safe for human consumption and have been granted Generally Recognized as Safe (GRAS) status by the United States Food and Drug Administration [34].

*Staphylococcus aureus* is a human Gram-positive pathogen involved in many diseases, including infective endocarditis, skin and soft tissue infections, osteomyelitis, septic arthritis, prosthetic device infections, pulmonary infections, gastroenteritis, meningitis, toxic shock syndrome, and urinary tract infections, and is often associated with biofilm formation [35,36]. A biofilm is a thin layer of microorganisms that forms on the surface of a solid substrate, such as medical devices [37]. Biofilms are prevalent forms of microbial life and can be found in various environments, causing sanitary problems and economic losses in the food industry [38]. *S. aureus* biofilms are the etiological agent of many recurrent infections with indwelling medical devices [35]. In the food industry, biofilm-forming species appear in factory environments, and they develop biofilm structures causing food contamination that can be pathogenic to humans [39]. Biofilm formation is a complex and dynamic process influenced by multiple factors and can change over time. In biofilms, the microorganisms communicate with each other and coordinate their activities using quorum sensing (QS) and the related soluble molecules, named autoinducers. This cell–cell communication process allows the biofilm to function as a single, coordinated, multi-cellular organization [40] made by one or more bacterial species. Since biofilms decrease the antibiotic diffusion rate within the bacterial population, they are difficult to control, leading to progression to chronic infection and disease [41,42]. Moreover, Gram-negative and Gram-positive bacteria can form complex multispecies biofilms wherein the QS of each component controls different physiological activities, including symbiosis, virulence, competence, conjugation, antibiotic resistance, and motility [43]. For instance, methicillin-resistant *Staphylococcus aureus* (MRSA) and *Pseudomonas aeruginosa*, a Gram-negative bacterium, compete for prevalence in the airways of cystic fibrosis patients, resulting in antibiotic resistance and exacerbation of the disease [44]. 

MRSA is a serious public health concern because of biofilm-associated infections [45,46,47,48,49]. In MRSA, QS is regulated by the accessory gene regulator (*agr* locus) and the production of autoinducing peptides (AIPs) that allow the bacterium to coordinate gene expression in response to changes in population density [50]. The *agr* system upregulates virulence factors involved in endocarditis, skin and soft tissue infections [51], hospital-acquired pneumonia [52], septic arthritis, and osteomyelitis [53]. AgrCA is a histidine kinase two-component system. Upon AIP-mediated activation, AgrA activates transcription from the P2 promoter, resulting in auto-feedback regulation [54] and increased expression of *agrA* and *rnaIII* genes, which encode regulatory effectors directly involved in biofilm formation and development [55]. AgrA is fully expressed in biofilm [56] and participates in different stages of biofilm formation, such as attachment, cell–cell adhesion, and detachment [57]. As a member of the LytR family, AgrA is a transcription factor characterized by the common structural motif known as the helix-turn-helix (HTH) domain, which is involved in DNA binding. Different selective inhibitors of AgrA DNA binding have recently been reported as a novel strategy to promote host defence [58]. Increasing consideration has been directed toward target-based screening for natural anti-biofilm agents [38,59]. Phenolics, essential oils, terpenoids, lectins, alkaloids, polypeptides, and polyacetylenes show anti-biofilm activity [60]. In our previous work, a labdane diterpenoid from *Salvia tingitana* showed synergistic activity with clindamycin and possible interference with *S. aureus* QS, probably due to interaction with the AgrA response regulator [61]. 

Abietane-type diterpenes and their derivatives have been tested against *S. aureus* biofilms [62]. Nakagawa et al. reported CA and carnosol extracted from rosemary as potent and specific inhibitors of *S. aureus* QS in clinical strains isolated from patients with atopic dermatitis, acting on *rnaIII* and *psmα* gene expression [63].

This study aimed to evaluate the waste biomass from agricultural cultivation and harvesting practices of the local *S. rosmarinus* ecotype, namely “Eretto Liguria”, as a source of valuable bioactive compounds. For this purpose, chemical profiling of the methanolic extract of this biomass was performed. Given the growing consideration directed toward selective inhibitors of *S. aureus* AgrA DNA binding [58,64,65,66,67,68,69], interference with QS signaling of MRSA by the most abundant compounds was explored. Moreover, CA was evaluated as an anti-biofilm agent during biofilm formation and species-specificity against MRSA was checked. Finally, in silico analysis was performed to hypothesize a possible binding mode of the selected compound with the AgrA active site.

## 2. Results

### 2.1. Qualitative Profiling of the Rosemary Extract

**Figure 1:**
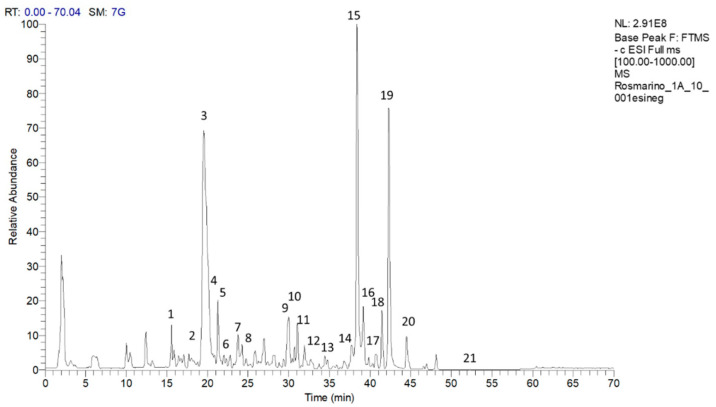
Chromatogram of compounds in the methanolic extract of the rosemary “Eretto Liguria” ecotype. Peak data are shown in Table 1. The peaks of the identified metabolites are annotated with numbers. Key: **1**: caffeic acid; **2**: isorhamnetin–3–*O*-hexoside; **3**: apigenin–7–*O*–glucoside; **4**: homoplantaginin; **5**: dihydrorabdosiin; **6**: rosmarinic acid; **7**: rabdosiin; **8**: isorhamnetin; **9**: rosmanol isomer; **10**: rosmanol isomer/epirosmanol; **11**: rosmanol isomer; **12**: rosmarinic acid isomer; **13**: rosmanol isomer; **14**: hydroxy–octadecatrienoic acid; **15**: carnosol; **16**: rosmadial; **17**: rosmadial isomer; **18**: hydroxy–octadecadienoic acid; **19**: carnosic acid; **20**: methyl carnosate; **21**: oleanolic acid.

A representative chromatogram of the methanolic extract of the rosemary “Eretto Liguria” ecotype is reported in Figure 1. The tentative identification of the metabolites was based on accurate mass measurements of the pseudomolecular [M–H]^–^ ions and their fragmentation pattern, compared with the literature. Table 1 shows the retention time (RT), quasimolecular ion [M-H]^–^ after negative ionization, and the MS/MS fragment ions. Diterpenoids, such as CA, carnosol, methyl-carnosate, rosmanol isomers, rosmadial, and rosmadial isomer, as well as the triterpenoid, oleanolic acid, were identified. Flavonoids such as isorhamnetin, isorhamnetin-3-*O*-hexoside, apigenin-7-*O*-glucoside, and homoplantaginin, and polyphenolic acids, such as rosmarinic acid, rosmarinic acid isomer, caffeic acid, rabdosiin, and dihydrorabdosiin, a tetramer of caffeic acid, were also present, along with hydroxy-octadecatrienoic acid and hydroxy-octadecadienoic acid. Finally, the ^1^H-NMR spectrum of the methanolic extract of the “Eretto Liguria” ecotype was also compared with the spectrum of the methanolic extract of *S. rosmarinus* Spenn. (upright habit) (Figure S1, Supplementary Materials).

### 2.2. Interference of Selected Compounds in Quorum Sensing Signaling of MRSA

**Figure 2:**
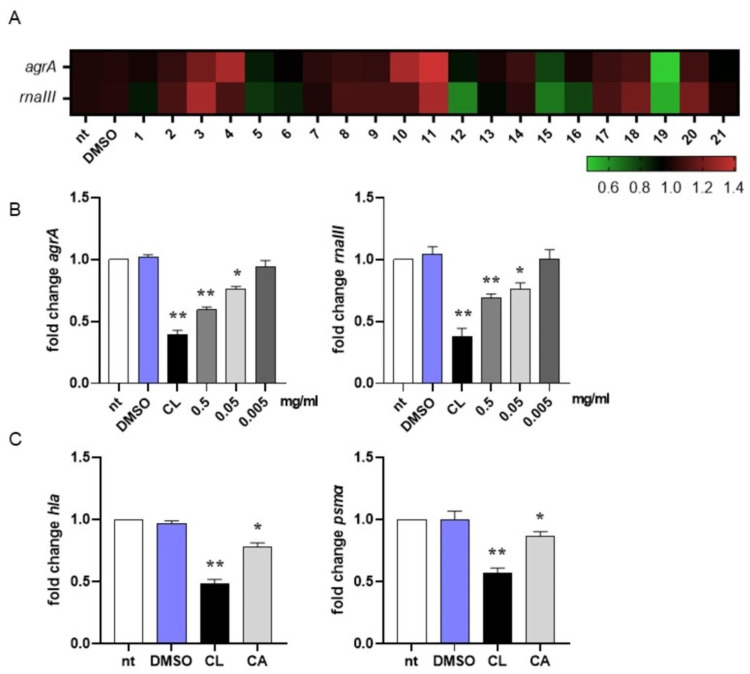
Interference in quorum sensing signaling of MRSA. (**A**) Heatmap illustrating differential gene expression over non-treated (nt) controls. MRSA cultures (10^6^ CFU/mL) were incubated at 37 °C for 16 h with the isolated compounds (0.5 mg/mL) reported in Table 1 or vehicle (DMSO 0.05% *v*/*v*). RNA was extracted and subjected to qRT-PCR to evaluate *agrA* and *rnaIII* gene expression. Expression values for each gene were normalized to the expression of the housekeeping gene *gyrB* and reported as fold change over non-treated (nt) samples. Increased gene expression is reported in red, and reduced gene expression is reported in green (color scale at bottom). Data were obtained from three independent experiments, each performed in duplicate. See Table 1 for sample identification. (**B**) MRSA cultures (10^6^ CFU/mL) were incubated at 37 °C for 16 h with carnosic acid (CA) at 0.5, 0.05, and 0.005 mg/mL or 0.25 µg/mL clindamycin (CL). Expression of *agrA* and *rnaIII* genes was assessed by qRT-PCR and normalized to the expression of the housekeeping gene *gyrB*. Data are reported as mean ± st err of three independent experiments, each performed in triplicate, and calculated as fold change relative to gene expression in non-treated (nt) samples. (**C**) MRSA cultures (10^6^ CFU/mL) were incubated at 37 °C for 16 h with CA at 0.05 mg/mL or 0.25 µg/mL clindamycin (CL). Expression of *hla* and *psmα* genes was assessed by qRT-PCR and normalized to the expression of the housekeeping gene *gyrB*. Data are reported as mean ± st. err. of three independent experiments, each performed in triplicate, and calculated as fold change relative to gene expression in non-treated (nt) samples. * denotes *p* < 0.05 vs. nt; ** denotes *p* < 0.02 vs. nt.

In MRSA, activation of the quorum sensing (QS) system controls virulence traits such as antibiotic resistance, transformation, and biofilm formation [79]. Since inhibition of QS disarms bacterial pathogenesis [80], in this study we evaluated the ability of the most abundant compounds of the methanolic extract of the rosemary “Eretto Liguria” ecotype (Table 1) to interfere with the QS system of MRSA by quantifying the expression of *agrA* and *rnaIII*, genes involved in QS activation and biofilm initiation, respectively [46]. Compounds were assessed at 0.5 mg/mL, the highest concentration obtained from the preparation procedure (see Section 4.3, Materials and Methods). As reported in Figure 2A, DMSO did not change gene expression compared to non-treated (nt) bacterial cultures at the concentration used to solubilize the compounds (0.05% *v*/*v*). Compounds **3** (apigenin-7-*O*-glucoside), **4** (homo-plantaginin (hispidulin 7-*O*-glucoside), **10** (rosmanol isomer/epi-rosmanol), and **11** (rosmanol isomer) increased the expression of *agrA* and/or *rnaII* genes compared to non-treated (nt) bacterial cultures, thus supporting QS activation. On the contrary, compounds **5** (dihydro-rabdosiin), **12** (rosmarinic acid isomer), **15** (carnosol), **16** (rosmadial), and **19** (CA) reduced the expression of QS-related genes. CA (**19**) was the most effective in reducing *agrA* and *rnaIII* gene expression. For this reason, in this study, we focused our attention on CA-mediated anti-virulence activity against MRSA.

We incubated MRSA cultures with CA at 0.5, 0.05, and 0.005 mg/mL to assess the lowest concentration required to inhibit QS-related genes. As reported in Figure 2B, 0.05 mg/mL was the lowest concentration of CA at which we still observed a significant reduction in *agrA* and *rnaIII* gene expression. For this reason, subsequent experiments were performed with CA at 0.05 mg/mL. To further confirm the ability of CA to inhibit QS in MRSA, we quantified the mRNA expression of *hla* and *psmα*, genes driving the toxic phenotype of the pathogen [46]. As reported in Figure 2C, CA at 0.05 mg/mL significantly reduced (around 20% compared with DMSO) the expression of these genes, supporting its role in shutting down the virulence of MRSA. Incubation with 0.25 μg/mL clindamycin, a protein synthase inhibitor recommended for the treatment of antibiotic-resistant *S. aureus* infections, reduced gene expression at a higher rate (around 50% vs. DMSO).

### 2.3. Carnosic Acid Has No Effect on the Viability of Bacteria and Eukaryotic Cells

**Figure 3:**
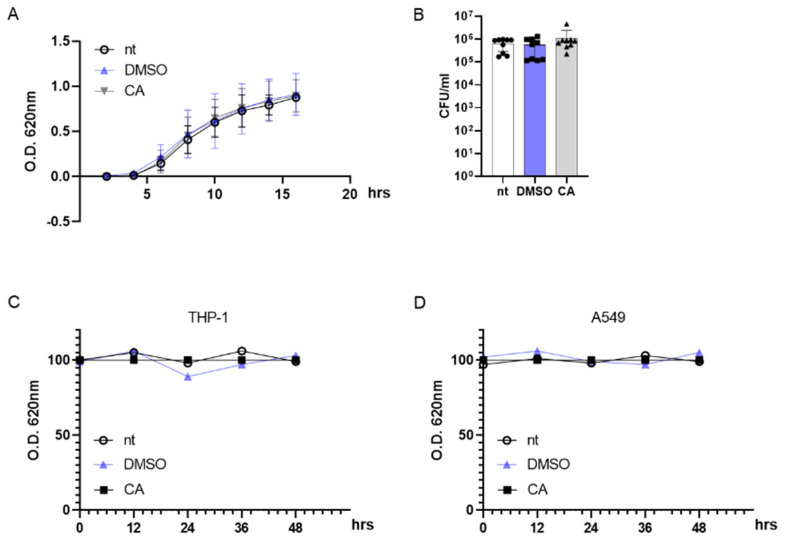
Carnosic acid (CA) exerts no effect on prokaryotic and eukaryotic cells. (**A**) MRSA (10^6^ CFU/mL) cultures were incubated at 37 °C with DMSO (0.05% *v*/*v*), CA (0.05 mg/mL), or were left untreated (nt). The growth kinetics were recorded for 16 h at 620 nm. (**B**) At the end of the incubation reported in (**A**), bacterial cultures were diluted and plated on agar media. Plates were incubated for 16 h at 37 °C and colonies were counted. (**C**,**D**) THP-1 and A549 cell lines were incubated with DMSO (0.05% *v*/*v*), CA (0.05 mg/mL), or were left untreated and cell viability was assessed by MTT assay at 0, 12, 24, 36, and 48 h. Data are reported as mean ± st err of three independent experiments, each performed in triplicate.

To rule out any possible effects of CA on MRSA survival, we incubated bacteria with 0.05 mg/mL CA and followed the bacterial growth kinetics by monitoring the optical density (OD) at 620 nm for 16 h. As reported in Figure 3A, incubation with DMSO at 0.05% or CA at 0.05 mg/mL had no effect on the exponential growth curve of MRSA, and the OD values did not statistically differ from those reported for non-treated (nt) MRSA cultures. At the same, 0.05 mg/mL CA did not alter the MRSA colony number on agar plates following 16 h of incubation (Figure 3B). Indeed, anti-virulence agents such as QS inhibitors shut down the pathogenicity of the bacteria with no reduction in cell viability, a mandatory condition for avoiding the occurrence of drug resistance [81].

Considering the possible use of CA as an anti-virulent MRSA agent in humans, we incubated differentiated THP-1 (macrophage-like) and A549 (lung adenocarcinoma) cell lines with 0.05 mg/mL CA. Indeed, MRSA is usually identified as an infectious agent in the airways [82]. As reported in Figure 3C,D, CA did not induce cytotoxic effects in eukaryotic cells with up to 48 h of incubation.

### 2.4. Carnosic Acid Interferes with Biofilm Formation by MRSA 

**Figure 4:**
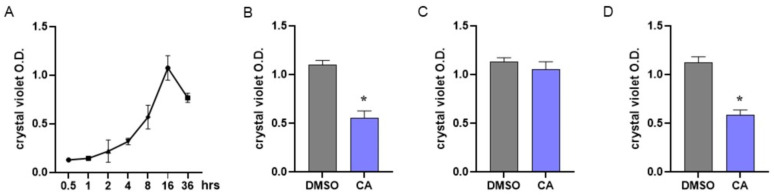
Effect of carnosic acid on MRSA biofilm. (**A**) To assess biofilm formation by MRSA, bacterial cultures were incubated under static conditions and biofilm formation was assessed at different time points by crystal violet staining. (**B**) MRSA cultures were treated with CA (0.05 mg/mL) and incubated at 37 °C under static conditions. Biofilm formation was evaluated 16 h later by crystal violet staining. (**C**) MRSA cultures were incubated for 8 h under static conditions and then treated with CA at 0.05 mg/mL. Cultures were incubated for 8 h more and biofilm formation was evaluated by crystal violet staining following 16 h of total incubation. (**D**) The experiments described in B were also performed with non-antibiotic-resistant *S. aureus*. Data are reported as mean ± st err of three independent experiments, each one performed in triplicate. * denotes *p* < 0.05 vs. DMSO.

In MRSA, biofilm formation is considered a significant virulence factor induced by QS activation [83]. To assess the dynamics of biofilm formation, we performed preliminary experiments by incubating MRSA under static conditions for 0–36 h and evaluating biofilm formation at different time points using crystal violet staining. As reported in Figure 4A, our experiments indicated that in MRSA cultures, bacteria adhere and form a biofilm starting 8 h following incubation; the number of adherent bacterial cells peaks following 16 h of incubation and then decreases. 

Anti-biofilm agents could act by interfering with biofilm formation or disrupting already mature biofilm. Considering the data reported in Figure 4A, MRSA cultures were immediately treated with CA at 0.05 mg/mL to assess the interference with biofilm formation (Figure 4B) or incubated with CA 8 h after the beginning of the culture to verify the disrupting effect (Figure 4C). Biofilms were assessed following 16 h of incubation. As reported in Figure 4B, incubation with CA starting from the beginning of the culture significantly prevented biofilm formation, whereas the addition of CA after 8 h of culture did not alter the already formed biofilm (Figure 4C). The effects of CA in preventing biofilm formation were also demonstrated with non-antibiotic-resistant *S. aureus* (Figure 4D).

To evaluate the specificity of CA as an anti-biofilm agent against MRSA, we performed similar experiments with *Pseudomonas aeruginosa*, a human pathogen usually associated with *S. aureus* in airway infections [84]. The QS system controls biofilm formation by *P. aeruginosa* as it does by *S. aureus*. However, the key molecular components involved in the QS of *P. aeruginosa* differ from those described in *S. aureus*, making QS inhibitors species-specific. As reported in Figure S2 (Supplementary Materials), in *P. aeruginosa* cultures, biofilm formation starts after 6 h incubation and peaks after 24 h incubation. The addition of CA did not interfere with biofilm formation, nor the ability to disrupt already mature biofilm, thus demonstrating anti-biofilm specificity against MRSA. 

### 2.5. Carnosic Acid Increases Intracellular Killing of MRSA

**Figure 5:**
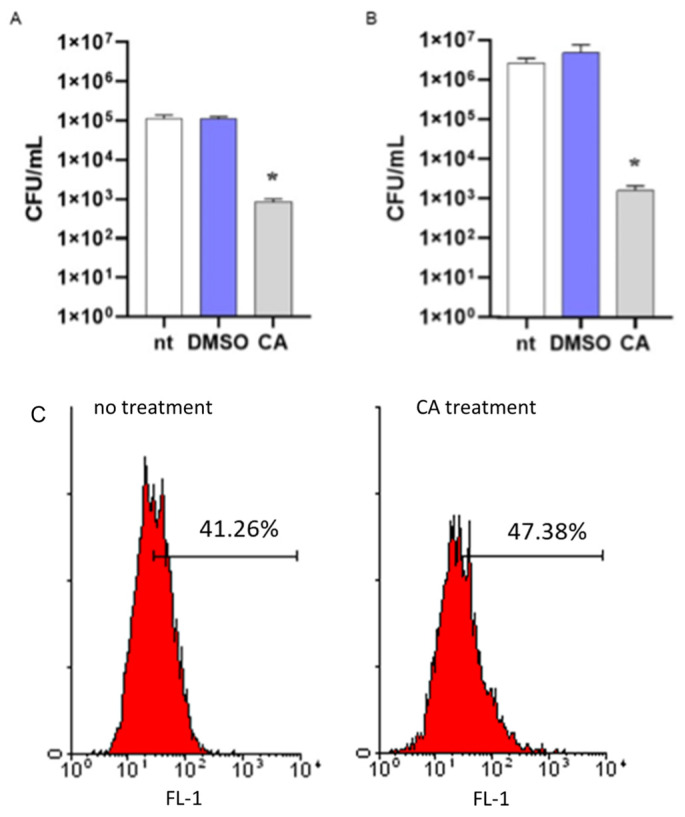
Effects of carnosic acid (CA) on MRSA intracellular killing. (**A**) MRSA cultures (10^6^ CFU/mL) were incubated for 16 h with CA (0.05 mg/mL), DMSO (0.05% *v*/*v*), or were left untreated (nt). Bacteria were then cultured with differentiated THP-1 (MOI 1:1) cells to assess phagocytic activity. Viable bacteria were enumerated by seeding the samples on agar plates. (**B**) The intracellular killing assay was also performed with non-antibiotic-resistant *S. aureus*. The experimental protocol was the same as described above. Data are reported as mean ± st err of three independent experiments, each performed in triplicate. * denotes *p* < 0.05 vs. nt. (**C**) To exclude any possible effects of CA in reducing the phagocytosis of THP-1 cells, fluorescent latex beads with 0.5 μm mean size were incubated (CA treatment) or not (no treatment) with CA, as described above. The phagocytic activity was determined by cytofluorimetric analysis on 10,000 collected events. FL-1, FITC fluorescent channel. The image is representative of 3 independent experiments.

To assess the role of CA in increasing the susceptibility of MRSA to intracellular killing, a mechanism of antibacterial immune defense in the host, we incubated bacteria with CA at 0.05 mg/mL or DMSO. After 16 h incubation, bacterial cultures were washed by centrifugation and incubated with differentiated THP-1 cells for 1 h to allow phagocytosis. Then, cells were washed, incubated with antibiotics to remove extracellular bacteria, and lysed. Intracellular bacteria were enumerated by seeding the samples in agar plates. As reported in Figure 5, MRSA cultures incubated with CA showed a reduced number of bacterial colonies as compared with cultures incubated with DMSO, thus demonstrating the ability of CA to reduce the survival of MRSA in the phagolysosomes of macrophage-like cells. The same data were obtained considering non-antibiotic-resistant *S. aureus* (Figure 5B). We performed a standard assay for phagocytosis to exclude any anti-inflammatory effects of CA in reducing the phagocytic activity of THP-1 cells. Latex fluorescent beads were incubated with or without CA, washed, and cultured with THP-1 cells as described for MRSA. Phagocyted beads were determined by flow cytometry analysis. As reported in Figure 5C, fluorescent signals related to phagocyted beads were comparable in THP-1 cells incubated with beads treated or not with CA.

### 2.6. NMR-Based Quantitative Analysis

Among the compounds in the methanolic extract of the rosemary “Eretto Liguria” ecotype identified by high-performance liquid chromatography (LC-MS/MS), CA (**19**) was the most effective in reducing *agrA* and *rnaIII* gene expression, and its content in the selected rosemary ecotype was then calculated (Figure S3, Supplementary Materials). The quantification was carried through qNMR using the 1D-NOESY sequence. The results showed that the CA content was 94.3 ± 4.3 mg/g of dry extract. 

### 2.7. Molecular Docking and Molecular Dynamics Simulations

**Figure 6:**
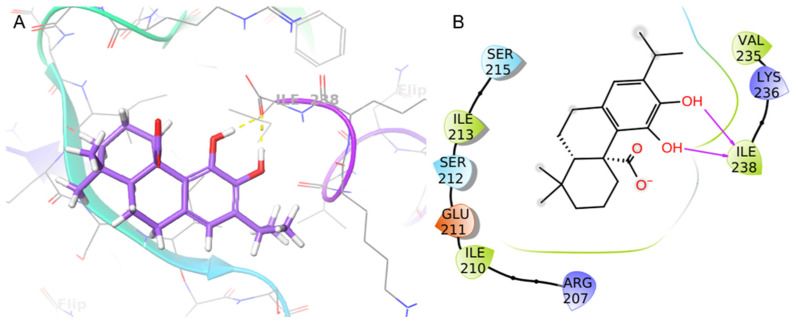
Binding pose (**A**) and interactions (**B**) of carnosic acid (CA) at the conserved AgrA active site. (**A**) The protein is reported as green-purple ribbons; the ligand is reported as purple capped sticks; H-bonds are presented as yellow dotted lines. (**B**) The ligand is surrounded by the protein residues represented as follows: the negatively charged residues are indicated in red, polar residues are in cyan, and hydrophobic residues are shown in green. H-bonds are depicted as purple arrows.

The binding mode of CA at the *S. aureus C*-terminus AgrA conserved region was investigated using Schrödinger Suite 2020-4. According to the literature, the grid box was centered at the *C*-terminal end of the LytTR domain, a site known to be important for DNA-binding activity [67]. In this region, the CA catechol moiety was bound to Ile238 with two H-bonds (1.69 Å and 1.68 Å) (Figure 6). This predicted binding mode was further stabilized by hydrophobic interactions with Ile210 and Ile213. CA achieved good binding score values (Glide binding energy −22.622 kcal/mol, docking score −3.686 kcal/mol). 4-Phenoxyphenol and 9H-xanthene-9-carboxylic acid, described in the literature [67] and recently incorporated in our work [61], showed similar binding energy values and docking scores in the same target binding pocket (4-phenoxyphenol: −22.191 kcal/mol, −4.471 kcal/mol; and 9H-xanthene-9-carboxylic acid: −20.841 kcal/mol, −4.546 kcal/mol). Moreover, CA demonstrated a similar binding pose as 4-phenoxyphenol, which was anchored to AgrA active site residues with its hydroxyl group. 

**Figure 7:**
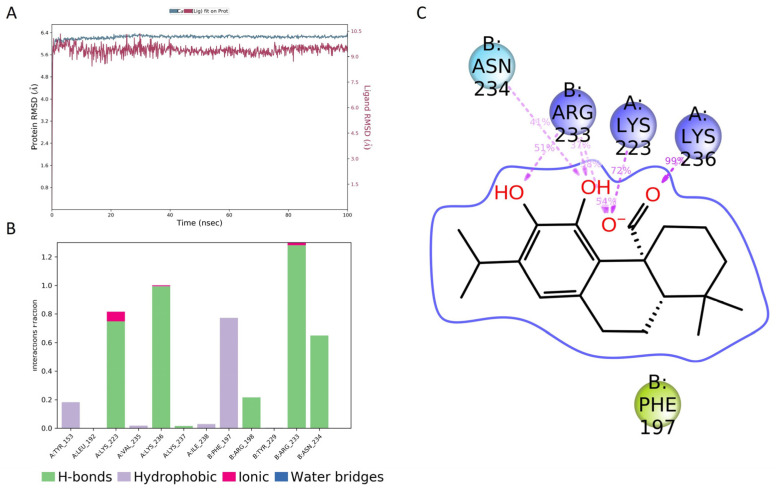
Molecular dynamics simulation results. (**A**) Root-mean-square deviation (RMSD) plot for the carnosic acid (CA)/AgrA complex along 100 ns molecular dynamics simulation related to Cα positions of residues belonging to the protein backbone (blue) and the ligand (purple). (**B**) Protein–ligand interactions (or ‘contacts’) plot for the CA/AgrA complex along 100 ns molecular dynamics simulation. Contacts are categorized into four types: hydrogen bonds, hydrophobic interactions, ionic bonds, and water bridges. (**C**) Ligand–atom interactions with the protein residues on chain A (Lys223 and Lys236) and on chain B (Arg233 and Asn234). Interactions that occurred more than 30.0% of the simulation time in the selected trajectory (0.00 through 100.00 ns) are shown.

The molecular dynamics (MD) of the CA/AgrA complex play a central role in the evaluation of the complex’s conformational stability. As shown in Figure 7A, the in silico investigation highlighted that CA was stable in the DNA-binding site during the whole 100 ns simulation. During the simulation time, several key interactions occurred with AgrA active site residues (Arg233, Asn234, Lys236). The H-bonds with Arg233 and Lys236 were maintained for the whole simulation time (Figure 7B). Other hydrogen bonds with Lys23 and Asn234 occurred more than 30.0% of the simulation time (Figure 7C). In addition, a hydrophobic interaction occurred with Phe197.

## 3. Discussion

Several studies have reported investigations of the phytochemical composition of rosemary extracts [11,12,85]. The methanolic extract of the rosemary “Eretto Liguria” ecotype was characterized by several abietane diterpenoids, together with other terpenoids, flavonoids, polyphenolic acids, hydroxy-octadecatrienoic acid, and hydroxy-octadecadienoic acid [10,11,71].

A significant quantity of CA (93.2 ± 4.4 mg/g of dry extract) was revealed, consistent with data reported for rosemary samples collected in the Mediterranean area [51]. CA has been isolated in a variety of plants, including *S. rosmarinus*, and it is also abundant in the harvested biomass [86]. This abietane diterpenoid is endowed with antimicrobial properties against various bacteria, fungi, and viruses, including *S. aureus* [31]. Nakagawa et al. [63] reported that CA and carnosol were able to reduce *rnaIII* and *psmα* gene expression. In the present study, we showed the ability of CA to reduce the expression of *agrA* and *rnaIII* virulence factors regulated by the *S. aureus* Agr system. In addition, the ability of CA to inhibit QS in MRSA was confirmed by quantification of the mRNA expression of *hla* and *psmα*, genes driving the toxic phenotype of the pathogen [49]. Moreover, our results demonstrated that CA exerted no effects on prokaryotic and eukaryotic cells, which is necessary to avoid the development of drug resistance. CA interfered with biofilm formation by MRSA, but it was not effective in destroying formed and mature biofilm, thus confirming its efficacy as a biofilm-preventing agent. Biofilm maturation leads to a more complex and organized structure; otherwise, antibiotics can trigger biofilm dispersal and release free-floating bacteria into the surrounding environment. Understanding the mechanisms involved in biofilm formation and how they can be manipulated is critical for the development of effective strategies to control biofilm-associated infections. Several plant extracts and compounds have been reported as inhibitors of MRSA biofilm formation [87,88,89,90,91,92]. Due to involvement of the *agr* system in controlling the expression of molecular effectors and biofilm development, targeting specific components of the system involved in signal transduction could be an excellent strategy to interfere with QS signaling [87]. In MRSA, the anti-virulence activity of QS inhibitors has been correlated with the reduced expression of *agrA* and *agr*-related genes [58,64,65,68,93]. 

Our experiments indicated that among the identified compounds (Table 1), only CA significantly reduced the expression of *agrA* and *rnaIII* genes (Figure 2A,B), thus interfering with the QS system in MRSA. The role of CA in shutting down the QS-mediated virulence of MRSA was further supported by our data reporting a reduction in biofilm formation (Figure 4B), a decrease in the expression of genes (*hla* and *psmα*, Figure 2C) coding for toxic traits of MRSA, and an increment in intracellular killing of MRSA phagocyted by macrophage-like cells (Figure 5). 

The inhibition of QS aims to disarm the virulence of pathogens without the selective pressure imposed by antibiotics. Besides the lack of bactericidal effects, QS inhibitors should exhibit species-specific anti-virulence activity to eventually avoid alterations in the prevalence of bacterial populations inside complex bacterial communities. It is well reported that antibiotic administration results in functional and structural microbiota alterations, namely dysbiosis, that affect the gut, skin, and lungs [94,95,96]. Particularly in the airways of cystic fibrosis patients, polymicrobial infections pose challenges in therapy because of the unknown consequences on microbial community members. Thus, the ecological networks based on interactions among QS systems change the response to antimicrobials and the relationships among components of the microbiota [44,97]. *S. aureus* and *P. aeruginosa* are the two most prevalent bacterial species in the lungs of cystic fibrosis patients, able to coexist as long as their relative abundance is maintained but capable of antagonism when one species prevails. Opposite to *S. aureus*, the QS system in *P. aeruginosa* uses acyl-homoserine lactone (AHL) molecules and four hierarchically interconnected circuits to regulate genes involved in virulence [98]. Therefore, local administration of QS inhibitors specific to a bacterial population should interfere with QS communications, deceiving the competitor. Our experiments demonstrated that CA selectively inhibited the molecular QS pathway in MRSA with no effects on *P. aeruginosa* biofilm formation (Figure S2, Supplementary Materials). These data support using this natural molecule as an anti-virulence agent in complex microbial infections even if further studies are needed to rule out the occurrence of resistance. 

In accordance with *agrA* and *rnaIII* reduced gene expression, we demonstrated that CA significantly decreased the mRNA transcripts specific for *hla* (gene encoding the α-hemolysin toxin, one of the major virulence factors in the pathogenesis of *S. aureus* infection) and *psmα* (gene encoding a toxin involved in bacterial invasion). The reduction in MRSA virulence following incubation with CA was also demonstrated by the increased susceptibility to intracellular killing activity, an immune response mechanism of the host. Indeed, the *agr* system regulates transcripts involved in resistance to the acid environment generated inside the phagolysosomes, resulting in the pathogen’s survival following phagocytosis. We demonstrated that by growing in the presence of CA, MRSA was efficiently killed compared with bacteria not treated with CA or treated with DMSO (Figure 5). Finally, incubation of planktonic cultures with CA inhibited biofilm formation, thus preventing one of the most severe virulence factors in MRSA infection (Figure 4B). We did not observe the effects of CA on mature biofilm (Figure 4C). Since the QS system acts not only during biofilm formation but also in biofilm maintenance and dispersion [99], we hypothesize that the lack of effect on mature biofilm is due to the inability of CA *per se* to penetrate the extracellular matrix of the biofilm. In conclusion, by disrupting the ability of MRSA to coordinate its QS, it may be possible to reduce the virulence of the bacteria and make them more susceptible to the host’s immune defence. In this study, we report that CA is specific for the QS of *S. aureus* but not that of *P. aeruginosa* and has no toxic effects on eukaryotic cells, making it a potential agent for clinical use by a topic application. 

QS inhibition has been proposed as an alternative to conventional antibiotics. Several studies on *S. aureus* virulence mechanisms recently underlined different inhibitors directed toward the transcriptional activator AgrA–DNA and *RNAIII* [100]. Moreover, different residues in the AgrA DNA-binding site have been reported for their remarkable role in QS regulation, particularly those reported by Leonard et al. [67]. Targeting AgrA in MRSA can be a strategy to specifically inhibit or disrupt the function of the AgrA response regulator [101]. This aim can be achieved by blocking the ability of AgrA to bind to DNA by inhibiting the enzyme activity required for AgrA to function. The molecular docking simulation revealed that CA bound with a pose like that of sclareol and manool [61], two labdane diterpenoids isolated from *S. tingitana*. Similar to manool, CA bound to Ile238, which has been demonstrated to be involved in AgrA DNA binding (Figure 6) [67]. This result was supported by 100 ns of molecular dynamics simulation, which showed that CA bound to the AgrA DNA-binding site (Figure 7). In addition, the molecular dynamics simulation revealed several crucial interactions that may govern the stability of the studied complex. The residues around Arg233 have been indicated as crucial in DNA binding [102]. These results pointed to the potential binding of CA with the response regulator AgrA as an obstacle in the *agr* system phosphorylation cascade or preventing AgrA binding to DNA. Our findings show that CA could be considered a potential anti-virulence agent against MRSA infections characterized by species-specific activity. Further research is needed to fully understand the anti-virulence effects of CA on clinical isolates and to develop effective strategies for its delivery.

## 4. Materials and Methods

### 4.1. Chemicals

Ultra-pure acetonitrile, water, methanol, and formic acid for LC-MS analysis were purchased from Romil Ltd. Pure Chemistry (Cambridge, UK). Solvents, deuterium oxide (D_2_O, 99.90% D), CD_3_OD (99.95% D), and 3-(trimethylsilyl)propionic-2,2,3,3-*d*_4_ acid sodium salt (TSP) were purchased from Sigma-Aldrich Chemical Company (Sigma-Aldrich, Milano, Italy).

### 4.2. Plant Material

Fresh aerial parts (1000.0 g) of waste biomass from agricultural cultivation of the *S. rosmarinus* Spenn. “Eretto Liguria” ecotype grown in Albenga (latitude 44°02′56.81″ N, longitude 8°12′46.86″ E, elevation 5 m.a.s.l) (Savona, Italy) were collected in July 2022. Fresh aerial parts of *S. rosmarinus* Spenn. (upright habit) grown in the experimental fields of CREA (Research Centre for Vegetable and Ornamental Crops, Corso Inglesi 508, 18038 Sanremo, Italy) in open air were also collected. The identification was performed by Dr. Andrea Copetta, according to the literature [1,103]. A voucher specimen (HMGBH.e/7219.2023.006) was deposited at the Herbarium of Giardini Botanici Hanbury (La Mortola, Ventimiglia, Italy).

### 4.3. Sample Collection and Preparation

Fresh biomass (5–10 cm at the top of plant shoots) including leaves and stems (1000 g) was frozen and lyophilized in a freeze dryer (Super Modulyo, Edwards, UK) for 48 h. 

The methanolic extract (9.1 g) for extraction was prepared by stirring 900 g of dried biomass with 1.0 L of CH_3_OH at 25 °C at 150 rpm for 24 h, followed by filtration through Whatman No. 4 paper. The extraction was repeated three times. The combined methanolic extracts were evaporated to dryness under reduced pressure and stored at 4 °C for further use. The isolated compounds (98% HPLC purity) (Table 1) were obtained from the methanolic extract following the procedure reported by Bisio et al. [104]. The extract for LC-MS/MS analysis was prepared by stirring 5 g of dried biomass with 100 mL of 100% of CH_3_OH at 25 °C at 150 rpm for 24 h, followed by filtration through Whatman No. 4 paper. The extraction was repeated three times. The combined extracts were evaporated to dryness under reduced pressure and stored at 4 °C for further use. Aliquots of dried rosemary extract (8 ± 0.3 mg) were dissolved in CH_3_OD to obtain stock solutions at 10 mg/mL. Samples were diluted at a concentration of 1 mg/mL and, after centrifugation at 13,000 rpm for 10 min, 600 µL of each were transferred into NMR tubes for further analysis, using TSP 0.1% as an internal standard for ppm calibration.

### 4.4. NMR Spectroscopy and Processing

The NMR experiments were performed on a Bruker Avance 600 spectrometer equipped with a 5 mm ATMA cryo-probe operating at 298 K and a SampleJet changer. TopSpin V3.2 software (Bruker Biospin, Wissembourg, France) was used for NMR data acquisition and processing, and its IconNMR module controlled the automation of acquisition, i.e., locking, tuning, matching, and shimming. ^1^H NMR spectra were recorded using a 1D-NOESY (noesygppr1d) pulse sequence with water signal suppression. The acquisition parameters were: 19K data points, 2782.7 Hz (11 ppm) spectral width, 4 dummy and 32 scans, a recycle delay of 4 s, and a fixed value for receiver gain for all samples. A ^1^H NMR spectrum of CA was acquired in MeOD to assign the chemical shifts of different protons. 

### 4.5. Qualitative Profiling of the Rosemary Extract

The extract was dissolved in CH_3_OH to obtain a final concentration of 1 mg/mL and subjected to LC-MS/MS analysis. A Luna^®^ C_18_ 150 × 2 mm, 3 µm (100 Å) column (Phenomenex^®^, Castel Maggiore, Bologna, Italy) was employed using H_2_O acidified by 0.1% formic acid *v*/*v* (solvent A) and CH_3_CN (solvent B) with the following linear gradient: solvent B from 5 to 100% in 50 min with subsequent reconditioning. The flow rate was set to 0.2 mL/min and the column oven was set to 40 °C. The LTQ XL™ Linear Ion Trap Mass Spectrometer (Thermo Fischer Scientific Inc., Darmstadt, Germany) was operated in negative ion mode coupled with the Thermo Scientific UltiMate 3000 UHPLC system. The identification of specialized metabolites was based on accurate MS values and MS/MS spectra and comparison with literature data [78] (Figure 1) (Table 1). 

### 4.6. Quantification of Carnosic Acid 

The ^1^H NMR spectra were acquired in triplicate. Abietane diterpenoids were then identified based on the comparison of their ^1^H NMR spectra to those in the Chenomx custom library. Quantitative analysis of NMR spectra was performed using NMRProcFlow 1.4.14 (INRA UMR 1332 BFP, Bordeaux Metabolomics Facility, Villenave d’Ornon, France) [105] following the method reported by Grimaldi et al. [106]. Briefly, corrections of phasing and baseline were performed manually for all spectra using TOPSPIN version 4.0.6. All spectra were calibrated using the internal standard at 0 ppm. Spectral area integration was performed by variable size bucketing using the online server NMRProcFlow. The analysis of ^1^H NMR chemical shifts of CA and the rosemary extract (Figure S3, Supplementary Materials) showed that the singlet at 6.32 ppm of CA was the only proton with a well-isolated chemical shift and could be used for quantitative study. All of the data needed were finally exported into a spreadsheet workbook using the “qHNMR” template. The calibration curve for CA was obtained at a concentration range from 10.0 µg/mL to 150.0 µg/mL. The linearity of the instrumental response in the analyzed concentration range was confirmed, as inferred by the following fitting curve parameters: y = 1773.8x + 11,266; R^2^ = 0.9996. The limit of detection (LOD) and limit of quantification (LOQ) were determined by serial dilution of CA, performed until the signal-to-noise ratio (S/N) reached the values of 3:1 and 10:1, respectively. The obtained values of LOD and LOQ were 2.3 µg/mL and 7.0 µg/mL, respectively.

### 4.7. Bacterial Strains and Growth Conditions

MRSA (strain number 33592; ATCC, LGC Standards; Milan, Italy), non-resistant *Staphylococcus aureus* subsp. *aureus* Rosenbach (ATCC 12600), and *Pseudomonas aeruginosa* (PAO1; CCUG241; Culture Collection University of Göteborg, Sweden) were maintained in Lysogeny broth or agar (LB; Fisher Scientific; Milan, Italy). Before experiments, MRSA or *P. aeruginosa* cultures were inoculated in fresh media (dilution 1:100) and incubated for 16 h at 37 °C. 

### 4.8. Total RNA Isolation and Quantitative RT-PCR

Overnight cultures of MRSA were diluted (1 × 10^6^ CFU/mL) in LB and incubated with DMSO at 0.05% *v/v*, the tested molecules, or 0.25 μg/mL clindamycin for 16 h, a condition previously reported to induce maximal expression of QS-related genes [72]. Cultures were centrifuged and total RNA was isolated and purified from the bacterial pellets using the GRS Total RNA Kit—Bacteria (GK16.0100; GRISP Research Solution, Porto, Portugal) following the manufacturer’s instructions. Purified RNA was subjected to DNase I treatment to remove contaminating DNA. The RNA yield and purity were assessed by measuring the absorbance, and only samples with a 260/280 nm ratio in the 1.8–2 range were used. cDNA was generated using the cDNA RT kit with an RNAse inhibitor (Applied Biosystems; Monza, Milan). Quantitative PCR (qRT-PCR) was performed using the SYBR green mixture (Bio-Rad, Segrate, Milano, Italy) to determine transcript levels of genes using the oligonucleotides listed in Table 2. The data were normalized to the expression of the housekeeping gene *gyrB* and reported as fold change over untreated cultures.

### 4.9. Bacterial Growth Kinetics

Overnight cultures of MRSA were collected, centrifuged, and suspended in LB broth at 10^6^ CFU/mL. Bacteria were dispensed in sterile 96-well microtiter plates and incubated with CA at 37 °C for 16 h. Bacterial growth kinetics were monitored by measuring the optical density at 620 nm at different time points using a Victor X2 Multilabel Microplate Reader (Perkin Elmer, Waltham, MA, USA). At the end of incubation, bacteria were diluted and cultured on agar plates. Plates were incubated at 37 °C for an additional 16 h. Bacterial colonies were enumerated. 

### 4.10. Cell Cytotoxicity 

The A549 (ATCC^®^ CCL-185TM) human lung carcinoma epithelial cell line was cultured in Dulbecco’s Modified Eagle’s Medium (DMEM) supplemented with 10% heat-inactivated fetal bovine serum (FBS) and 1% penicillin/streptomycin (all purchased from Thermo Fisher Scientific; Milan, Italy). The THP-1 human cell line (ATCC^®^ TIB-202TM) was maintained in RPMI 1640 supplemented with 10% FBS, HEPES (10 mM), sodium pyruvate (1 mM; Thermo Fisher Scientific), and β-mercaptoethanol (0.05 mM, Merck; Milan, Italy) and incubated at 37 °C with 5% CO_2_ in a humidified incubator. THP-1 cells were grown to a density of 1 × 10^5^ cells/mL. For differentiation to the macrophage phenotype, THP-1 cells (1 × 10^5^ cells/well) were treated with phorbol 12-myristate 13-acetate (PMA, 50 nM; Merck) and cultured for 48 h.

To assess cell cytotoxicity, we incubated cells with CA or DMSO for 16 h at 37 °C. Cell cultures were then treated with MTT (3-(4,5-dimethylthiazol-2-yL)-2,5-diphenyltetrazolium bromide, Merck) solution (5 mg/mL) for 4 h at 37 °C. Formazan crystals were solubilized in 100 μL of sodium dodecyl sulfate (SDS, 10% *w*/*v*) and HCl (0.01 N) and the absorbance was recorded 16 h later at 590 nm using a microplate reader (Victor X2 Multilabel Microplate Reader). 

### 4.11. Assessment of Anti-Biofilm Activities 

Overnight cultures of MRSA or *P. aeruginosa* were diluted 1:1000 in LB broth and 200 µL/well was seeded into 96-well polystyrene microtiter plates. Cultures were incubated at 37 °C under static conditions. To identify the time of incubation needed to obtain a mature biofilm, we incubated the plates for different time points, ranging from 30 min to 48 h. Then, the wells were emptied and washed three times with sterile PBS to remove planktonic cells. Adhering cells were stained with 150 μL of 0.1% (*w*/*v*) crystal violet (CV) solution for 15 min at room temperature. The plates were washed, air-dried, and CV was solubilized in 125 μL of 30% (*v*/*v*) glacial acetic acid per well. The optical density was measured at 570 nm using a microplate reader (Victor X2 Multilabel Microplate Reader).

To assess the effect of CA on preventing biofilm formation, we incubated bacterial cultures prepared as described above with CA, and biofilms were evaluated by crystal violet staining following 16 h of incubation for MRSA or following 36 h of incubation for *P. aeruginosa*. To observe the effects on already formed biofilms, we added CA following 8 h of incubation for MRSA or 6 h of incubation for *P. aeruginosa*. MRSA and *P. aeruginosa* were incubated for an additional 8 or 16 h, respectively. At the end of incubation, biofilms were evaluated by crystal violet staining. Bacteria incubated with DMSO were assigned 100% biofilm formation. 

### 4.12. Intracellular Bacterial Killing

For intracellular bacterial killing, MRSA was cultured with CA or DMSO at 0.05% *v*/*v* for 16 h at 37 °C. Control samples were cultured under the same conditions with no treatment. Bacteria were then washed and incubated with differentiated THP-1 cell cultures at a multiplicity of infection (MOI) of 1:1. Culture plates were centrifuged at 500× *g* for 3 min to facilitate cell–bacteria contact and then incubated at 37 °C in 5% CO_2_ for 1 h to allow phagocytosis. At the end of incubation, cells were treated with lysostaphin (2 mg/mL, 15 min; Merck) to kill extracellular bacteria. To enumerate the phagocyted bacteria, we disrupted the cell membranes using PBS/0.1% Triton X-100. The collected cell lysates were serially diluted and seeded on LB agar plates for 16 h at 37 °C. Bacterial colonies were enumerated. To rule out any possible interference of CA with the phagocytic activity of THP-1 cells, latex beads coated with FITC-labeled rabbit IgG (Item No. 500290, Cayman, distributed by Vinci-Biochem Srl, Vinci (FI), Italy) were incubated with CA as described above. Beads were washed and cultured with THP-1 cells. Cell cultures were washed, collected, and incubated with trypsin. Phagocyted beads were determined by cytofluorimetric assay using a BD-FACS Calibur (Becton Dickinson Rowa, Milano, Italy), acquiring 10,000 events. Flow cytometry analysis was performed using CellQuest software version 7.5.3 (Becton Dickinson). 

### 4.13. Molecular Docking and Molecular Dynamics Simulations

The crystal structure of the *S. aureus* AgrA LytTR domain (PDB code: 4G4K) [67] was obtained from the Protein Data Bank [107]. Missing side chains and hydrogens were added and optimized using the Protein Preparation Wizard embedded in Schrödinger Suite 2020-4 [108], and pH was set to 6.0 ± 1.0, optimizing the protonation states and the formation of disulphide bridges. Water molecules and glycerol were removed. The structure was then energy minimized using the OPLS3e force field to constrain heavy atoms. The AgrA active site has been defined as a common locus at the *C*-terminal end of the LytTR domain, a site known to be important for DNA-binding activity [67]. The chemical structure of CA was constructed using Maestro Build Panel and energetically minimized with the LigPrep module using the OPLS3e force field [109]. All possible tautomers and protonation states at pH 6.0 ± 1.0 were considered. The Receptor Grid Generation tool in the Glide module (part of Schrödinger Suite 2020-4) was used to set up a 35 Å^3^ grid box placed at the AgrA active site (Ser231, Val232, Arg233, Asn234, Lys236, Lys237, and Ile238). The grid was centred at x = 38.665, y = 33.270, and z = 57.085. The molecular docking simulation was carried out with Glide in SP/XP mode. The two approaches resulted in agreement, proposing similar binding modes. The docked pose of CA bound to AgrA was submitted to the molecular dynamics simulation using Desmond Molecular Dynamic [108]. The starting complex was prepared by System Builder in Desmond. A cubic box with a 10 Å buffer distance was set; the TIP3P water model for solvation and OPLS3e force field were employed, adding 6 Cl^−^ ions to obtain electroneutrality. After the minimization process using the Minimization tool in Desmond, an MD simulation of 100 ns at 298.1 K was performed, using a recording interval of 100 ps and ensemble class NPT (1.01 bar).

## 5. Conclusions

The rosemary “Eretto Liguria” ecotype was characterized by high content of the abietane diterpenoid, CA. CA was shown to downregulate *agrA*-related virulence gene expression, like *rnaIII*, one of the key effectors of QS. Moreover, inhibition of biofilm formation by MRSA occurred when CA was added at the beginning of the culture, demonstrating its potential as a preventative agent against biofilm formation. CA was demonstrated to be species-specific against MRSA without any effect on *P. aeruginosa*. Finally, molecular docking and molecular dynamics simulations showed a possible interaction pattern of CA with the AgrA response regulator active site. Further research is needed to test solvent mixtures with less impact [110] and to develop effective strategies for disrupting MRSA biofilm formation using natural compounds.

## Figures and Tables

**Table 1:** Compounds identified in the methanolic extract of rosemary “Eretto Liguria” ecotype identified by high-performance liquid chromatography (LC-MS/MS) in the negative ionization mode.

Peaks	Compound	Molecular Formula	RT (min)	[M-H]^−^ (*m/z*)	Base Peak	ESI-MS/MS Ions (*m/z*)	MSI Status ^*a*^	Reference
**1**	Caffeic acid	C_9_H_8_O_4_	15.98	179.0338	135	135	2	[70]
**2**	Isorhamnetin-3-*O*-hexoside	C_22_H_22_O_12_	19.14	477.1034	315	462, 315, 300	2	[71]
**3**	Apigenin-7-*O*-glucoside	C_21_H_20_O_10_	19.85	431.0980	269	269	2	[71]
**4**	Homoplantaginin (hispidulin 7-*O*-glucoside)	C_22_H_22_O_11_	20.30	461.1078	284	284, 299, 446	2	[72]
**5**	Dihydrorabdosiin	C_36_H_32_O_16_	21.16	719.1615	539	539, 359	2	[73]
**6**	Rosmarinic acid	C_18_H_16_O_8_	21.26	359.0761	161	161, 197, 179	2	[71]
**7**	Rabdosiin	C_36_H_30_O_16_	24.34	717.1450	519	519	2	[73]
**8**	Isorhamnetin	C_16_H_12_O_7_	24.50	315.0499	300	300, 315	2	[70]
**9**	Rosmanol isomer	C_20_H_26_O_5_	30.30	345.1696	301	301, 283	2	[71]
**10**	Rosmanol isomer/Epirosmanol	C_20_H_26_O_5_	31.32	345.1696	283	283	2	[71]
**11**	Rosmanol isomer	C_20_H_26_O	32.02	345.1696	301	301, 283	2	[71]
**12**	Rosmarinic acid isomer	C_18_H_16_O_8_	32.11	387.2023	205	205	2	[71]
**13**	Rosmanol isomer	C_20_H_26_O	34.31	345.1696	301	301, 283	2	[74]
**14**	Hydroxy-octadecatrienoic acid	C_18_H_30_O_3_	38.14	293.2118	275	275, 231, 235	2	[75]
**15**	Carnosol	C_20_H_26_O_4_	38.17	329.1747	285	285	2	[71]
**16**	Rosmadial	C_20_H_24_O_5_	39.66	343.1540	299	299, 315	2	[76]
**17**	Rosmadial isomer	C_20_H_24_O_5_	40.33	343.1540	299	299, 315	2	[74]
**18**	Hydroxy-octadecadienoic acid	C_18_H_32_O_3_	40.77	295.2276	277	277, 233	2	[77]
**19**	Carnosic acid	C_20_H_28_O_4_	42.02	331.1903	287	287	1	[71]
**20**	Methyl carnosate	C_21_H_30_O_4_	44.48	345.2060	301	301, 286	2	[71]
**21**	Oleanolic acid	C_30_H_48_O_3_	50.19	455.3519	407	407, 189	2	[71]

^*a*^ MSI level of identification according to Sumner et al. [78].

**Table 2:** Sequences of the oligonucleotides used in the qPCR experiments.

Gene	Oligonucleotide
*gyrB*	fw 5′-CAAATGATCACAGCATTTGGTACAG-3′ rv 5′-CGGCATCAGTCATAATGACGAT-3′
*rnaIII*	fw 5′-TTCACTGTGTCGATAATCCA-3′ rv 5′-TGATTTCAATGGCACAAGAT-3′
*agrA*	fw 5′-GCACATACACGCTTACAATTGTTG-3′ rv 5′-ACACTGAATTACTGCCACGTTTTAAT-3′
*hla*	fw 5′-ATGGATAGAAAAGCATCCAAACA-3′ rv 5′-TTTCCAATTTGTTGAAGTCCAAT-3′
*psmα*	fw 5′-TATCAAAAGCTTAATCGAACAATTC-3′ rv 5′-CCCCTTCAAATAAGATGTTCATATC-3′

fw: forward; rv: reverse.

## Data Availability

The data presented in this study are available in the article or Supplementary Material.

## Supplementary Materials

The following supporting information can be downloaded at: https://www.mdpi.com/article/10.3390/plants12213679/s1, Figure S1: Comparison between the 1H NMR spectrum of the methanolic extract of “Eretto Liguria” rosemary ecotype (red) and the spectrum of *S. rosmarinus* Spenn. (upright habit), grown in the experimental fields of CREA (Research Centre for Vegetable and Ornamental Crops, Corso Inglesi 508, 18038 Sanremo, Italy), in the open air (blue); Figure S2: Effect of carnosic acid (CA) on *Pseudomonas aeruginosa* biofilm; Figure S3: ^1^H NMR spectra of carnosic acid (CA) (red) and “Eretto Liguria” rosemary ecotype extract (blue).
