# Supplementary material for: Anti-Biofilm Activity of Carnosic Acid from Salvia rosmarinus against Methicillin-Resistant Staphylococcus aureus
Source: Plants (Basel). 2023 Oct 25;12(21):3679. doi: 10.3390/plants12213679 (PMC10647425; doi:10.3390/plants12213679)
Supplement: Supplementary file 1 [file plants-12-03679-s001.zip › plants-2621984-Supplementary.pdf]

plants

Supplementary Materials

Anti-biofilm activity of carnosic acid from *Salvia rosmarinus* against Methicillin-resistant *Staphylococcus aureus*

Valeria Iobbi [1], Valentina Parisi [2], Giulia Bernabè [3], Nunziatina De Tommasi [2], Angela Bisio [1,*], Paola Brun [3]

[1] University of Genova, Department of Pharmacy, Viale Cembrano 4, 16148 Genova, Italy
[2] University of Salerno, Department of Pharmacy, Via Giovanni Paolo II 132, 84084 Salerno, Italy
[3] University of Padova, Department of Molecular Medicine, Via Gabelli 63, 35121, Padova, Italy
* Correspondence: angela.bisio@unige.it

Supplementary Figures:

Figure S1. Comparison between the ^{1}H NMR spectrum of the methanolic extract of "Eretto Liguria" rosemary ecotype (red) and the spectrum of *S. rosmarinus* Spenn. (upright habit), grown in the experimental fields of CREA (Research Centre for Vegetable and Ornamental Crops, Corso Inglesi 508, 18038 Sanremo, Italy), in the open air (blue).
Figure S2. Effect of carnosic acid (CA) on *Pseudomonas aeruginosa* biofilm.
Figure S3. ^{1}H NMR spectra of carnosic acid (CA) (red) and "Eretto Liguria" rosemary ecotype extract (blue).

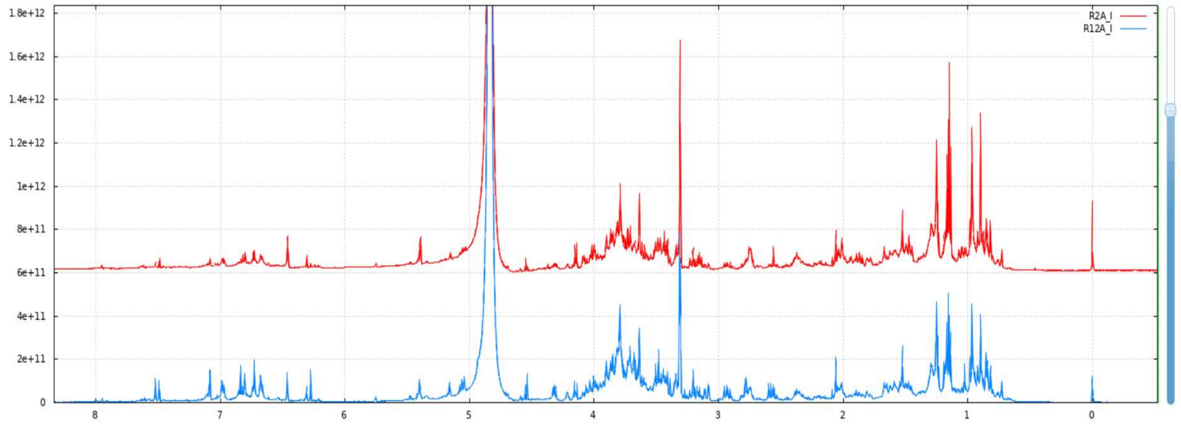

Figure S1. Comparison between the ^{1}H NMR spectrum of the methanolic extract of "Eretto Liguria" rosemary ecotype (red) and the spectrum of *S. rosmarinus* Spenn. (upright habit), grown in the experimental fields of CREA (Research Centre for Vegetable and Ornamental Crops, Corso Inglesi 508, 18038 Sanremo, Italy), in the open air (blue).
The complexity of the spectra is due to the overlapping of the signals.

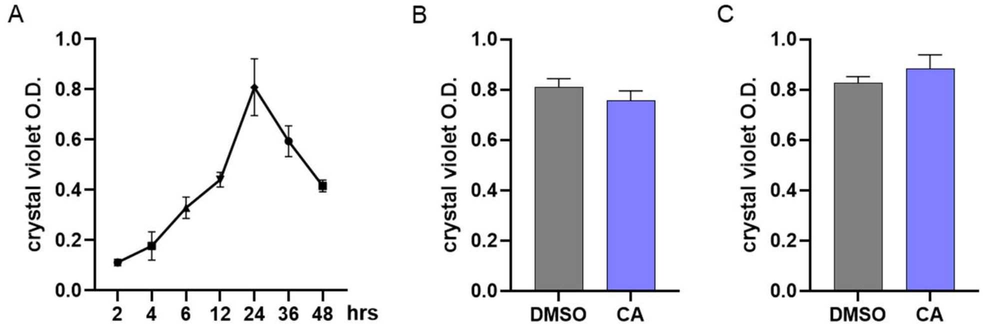

Figure S1. Effect of carnosic acid (CA) on *Pseudomonas aeruginosa* biofilm.

(A) To assess biofilm formation in *P. aeruginosa*, bacterial cultures were incubated under static conditions and biofilm was assessed at different time points by crystal violet staining. (B) *P. aeruginosa* cultures were added with CA (0.05 mg/mL) and incubated under static conditions. Biofilm formation was evaluated 24 h later by crystal violet staining. (C) *P. aeruginosa* cultures were incubated for 6 h under static conditions and then added with CA 0.05 mg/mL. Cultures were incubated for 18 h more and biofilm formation was evaluated by crystal violet staining following 24 h of total incubation. Data are reported as mean±st err of three independent experiments, each one performed in triplicate.

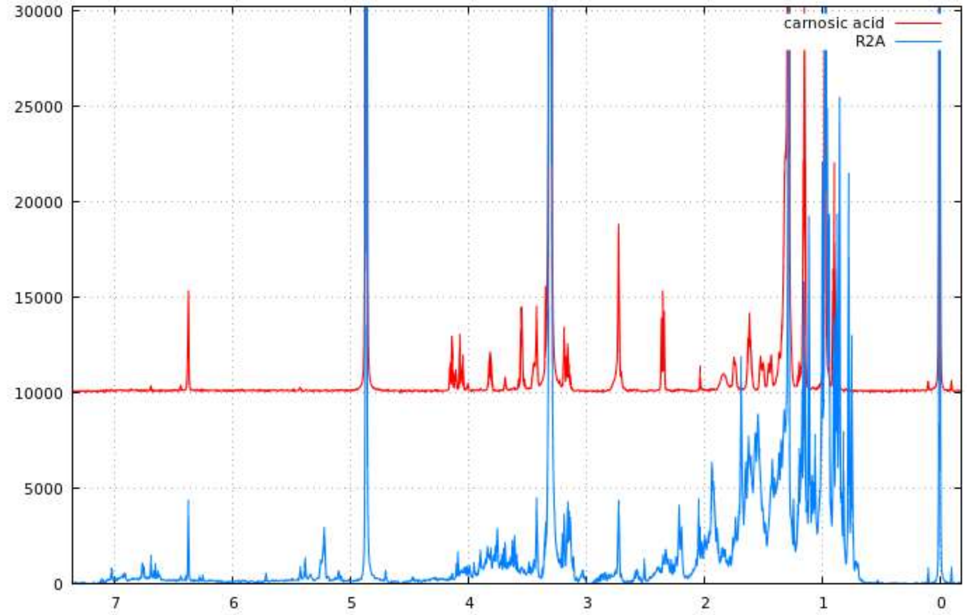

Figure S3. ^{1}H NMR spectra of carnosic acid (CA) (red) and "Eretto Liguria" rosemary ecotype extract (blue).

The complexity of the spectra is due to the overlapping of the signals.
